# Response to Emerging Infection Leading to Outbreak of Linezolid-Resistant Enterococci

**DOI:** 10.3201/eid1307.070019

**Published:** 2007-07

**Authors:** Marion A. Kainer, Rose A. Devasia, Timothy F. Jones, Bryan P. Simmons, Kelley Melton, Susan Chow, Joyce Broyles, Kelly L. Moore, Allen S. Craig, William Schaffner

**Affiliations:** * Tennessee Department of Health, Nashville, Tennessee, USA; † Centers for Disease Control and Prevention, Atlanta, Georgia, USA; ‡ Methodist University Hospital, Memphis, Tennessee, USA; § Vanderbilt University School of Medicine, Nashville, Tennessee, USA

**Keywords:** Drug resistance, bacterial, enterococcus, oxazolidinones, antimicrobial agents, disease transmission, horizontal, drug utilization review, methicillin resistance, *Staphylococcus aureus*, research

## Abstract

Linezolid was approved in 2000 for treatment of gram-positive coccal infections. We performed a case-control study during a hospital outbreak of linezolid-resistant enterococci (LRE) infections, comparing cases of LRE infection (cases) with linezolid-sensitive enterococci infections (controls). Nasal and perirectal swab samples were obtained from all patients in a 1-day point-prevalence survey. We examined antimicrobial drug use and calculated the defined daily dose of linezolid per 1,000 patient-days. Fifteen LRE cases were identified (13 *Enterococcus faecalis* and 2 *E*. *faecium*); 7 were vancomycin-resistant. Compared with controls, case-patients had increased in-hospital mortality rates and lengths of stay. Multivariate analysis identified independent predictors of LRE infection: prior cultures positive for methicillin-resistant *Staphylococcus aureus* (adjusted odds ratio [AOR] 27), hospitalization duration before index culture (AOR 1.1 per day), and duration of preceding linezolid therapy (AOR 1.1 per day). Linezolid exposure and patient-to-patient transmission appear to be responsible for LRE infections, an important emerging hospital problem.

## Abstract 2

These bacteria have emerged as a hospital problem that appears to be caused by both linezolid exposure and patient-to-patient transmission.

Enterococci are common inhabitants of the human gastrointestinal tract. Although >40 enterococcus species exist, nosocomial infections are primarily caused by *Enterococcus faecalis* and *E. faecium* (*1*). Enterococcal infections are the third most common cause of nosocomial infection in intensive care units (ICUs), and multidrug-resistant enterococcal infections have been associated with higher hospitalization costs and a higher number of related deaths (*2*,*3*).

Linezolid, 1 of the oxazolidinone class of antimicrobial drugs, inhibits bacterial protein synthesis by binding to the 50S subunit of 23S rRNA. In April 2000, linezolid was approved in the United States and has been heavily marketed to treat methicillin-resistant *Staphylococcus aureus* (MRSA) and vancomycin-resistant enterococci (VRE) infections (*4*,*5*). Although more expensive than vancomycin, linezolid does not require testing for adequate serum drug concentrations or dosing adjustment for renal or hepatic insufficiency (*6*), and it has been regarded by some healthcare providers as more effective than vancomycin in treating nosocomial pneumonia and MRSA skin and soft tissue infections (*7*–*9*). Most reports of linezolid-resistant enterococci (LRE) have been individual cases or small case series (*10*–*20*) or have specifically described linezolid-resistant and vancomycin-resistant *E. faecium* (LRVRE) (*17*–*22*). We describe a large hospital outbreak of LRE infections.

Hospital A is an urban, 500-bed, adult inpatient, teaching facility with surgical, transplant, and medical ICUs in a city of ≈850,000 persons. Community-associated MRSA is an important emerging pathogen in that city (*23*,*24*). At hospital A in 2004, 154 MRSA and 29 VRE blood culture isolates were identified; compared with results for 1997, these are increases of 428% and 725%, respectively (Figure 1). Linezolid became available in hospital A in April 2000, but it was restricted for use by infectious disease and critical care physicians only, some of whom believed it provided an advantage over vancomycin for treatment of MRSA, especially pulmonary MRSA (*8*,*9*). In February 2005, hospital A’s infection-control staff contacted the Tennessee Department of Health after isolating LRE in the blood culture of a ventilated patient. Within a week, surveillance cultures identified a second patient with LRE in the same unit. We undertook an investigation to characterize the epidemiology of LRE, to determine risk factors for emergence of linezolid resistance in enterococci among patients previously infected or colonized with linezolid-sensitive enterococci (LSE), and to determine outcomes associated with LRE infection. This investigation was approved by the institutional review board of hospital A.

**Figure 1:**
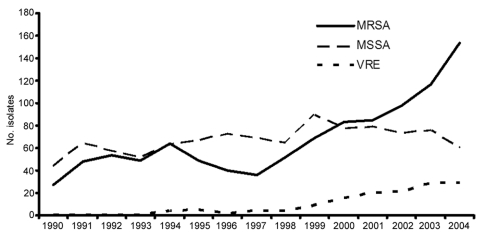
No. nonduplicate blood-culture isolates of methicillin-resistant *Staphylococcus aureus* (MRSA), methicillin-sensitive *S. aureus* (MSSA), and vancomycin-resistant *Enterococcus faecalis* and *E. faecium* (VRE) per year, hospital A, Tennessee, 1990–2004.

## Methods

### Epidemiology

Cases were identified by manually reviewing hospital A’s microbiology susceptibility testing reports related to all *E. faecium* and *E. faecalis* isolates for January 2004 through February 2005. Patients for whom a clinical isolate of LRE had been identified during the study period were selected for the study. For each case, 4 randomly selected hospitalized control subjects with LSE were identified by using hospital microbiology reports; no matching was performed. The index hospitalization was defined as the hospital admission during which LRE or LSE had been identified. Trained staff performed chart reviews by using standard questionnaires to determine demographics, hospital course, immunocompromising conditions, instrumentation during the index hospitalization, and history of hospitalization and inpatient antimicrobial drug exposure during the 12 months preceding the index isolate. Instrumentation was defined as receipt of Foley catheterization, chest tube, Swan-Ganz catheterization, mechanical ventilation, dialysis catheter, central line, arterial line, peripherally inserted central catheter, permanent central venous catheter, balloon pump, or intraabdominal or other surgery. Immunocompromising conditions were defined as the presence of leukemia or nonskin cancer, chronic renal failure, requiring dialysis, diabetes, HIV infection, pancreatitis, steroid use of >10 mg for >5 days, or solid organ or stem cell transplantation. Mortality was defined as patient death during the index hospitalization. Critical care areas were defined as the ICUs, extended postoperative holding area (overflow ICU), coronary care unit, and ventilator rehabilitation unit.

Specimen information for clinical isolates of *E*. *faecium*, *E*. *faecalis*, *S*. *aureus*, and *S*. *epidermidis* was obtained for the index hospitalization and for the preceding 12 months. Isolates from surveillance cultures (not illness-associated) were not included. Invasive clinical isolates were defined as isolates from any of the following sources: blood; bone; cerebrospinal, joint, pericardial, peritoneal, or pleural fluid; surgical specimen or aspirate; or any other normally sterile site. Noninvasive isolates included isolates from sputum, urine, or wounds. We performed a subset analysis comparing linezolid-sensitive and vancomycin-resistant enterococci (LSVRE) and LRVRE infections (case-control study II). After the initial random selection of controls with LSE infections, we selected additional controls with LSVRE to obtain a 1:4 ratio of LRVRE-infected case-patients to LSVRE controls.

### Point-Prevalence Survey

A 1-day point-prevalence culture survey for *E*. *faecalis, E*. *faecium*, and *S*. *aureus* was performed in hospital A. On March 28, 2005, all patients hospitalized in hospital A were asked if they would give informed consent for the collection and culture of nasal and perirectal swab specimens; specimens were obtained from all patients who gave consent. Identification and antimicrobial drug susceptibilities of nasal and perirectal swab specimen cultures were performed in hospital A’s microbiology laboratory.

### Laboratory Studies

Hospital A used the Dade Microscan Walkaway 96 (Diamond Diagnostics, Holliston, MA, USA) SI Pos Combo 21 for all species identification and susceptibility testing of gram-positive clinical isolates (*25*). Linezolid resistance was confirmed at the Centers for Disease Control and Prevention (CDC, Atlanta, GA, USA) by use of the broth-dilution method; the MIC was >16 µg/mL for all isolates tested. For the point-prevalence survey, hospital A microbiology staff plated nasal swabs specimens onto mannitol-salt agar plates; linezolid (30 µg) and oxacillin (1 µg) disks were placed in the first quadrant to screen for LRSA and MRSA, respectively. Perirectal swabs samples were plated directly onto 2 bile-esculin plates, 1 of which contained 6 µg vancomycin/mL to screen for VRE. A linezolid disk was placed on the heavy inoculum on the plate without vancomycin to screen for LRE.

Pulsed-field gel electrophoresis (PFGE) subtyping was performed at the Tennessee Department of Health laboratory on available LRE isolates (clinical [3], surveillance [3], and environmental [3]) from hospital A. The PulseNet (CDC) standardized protocol for *Listeria monocytogenes* (*26*) was used for DNA preparation. Specific conditions adapted for this application included separate 5-h digestions with 100 U *Sma*I and 100 U *Apa*I restriction endonucleases and 18-h electrophoresis, using a CHEF Mapper (Bio-Rad Laboratories, Hercules, CA, USA) programmed for a molecular weight range of 25–350 kb, an initial switch time of 2.0 s, and a final switch time of 20.0 s. The PulseNet size standard, *S.* Braenderup strain H9812 (ATCC BAA-664) (*27*), was used as a size marker. PFGE fingerprint types were assigned by using the criteria of Tenover et al. (*28*).

By using laboratory information system data, we determined the number of MRSA, methicillin-sensitive *S. aureus*, and VRE isolates cultured from hospital A inpatient blood samples from January 1990 through December 2004. Repeat isolates from the same patient within 30 days were excluded.

### Antimicrobial Drug Usage

The defined daily dose (DDD) of linezolid was identified as 1.2 g/day. We reviewed hospital A’s pharmacy purchase data for linezolid for October 2001 through February 2005 and calculated DDD/1,000 patient-days; intravenous linezolid usage was analyzed by patient location (ICU or non-ICU). Oral linezolid use was not tracked by patient location. Hospital A’s antimicrobial drug–prescribing restrictions were reviewed. Hospital A pharmacy staff conducted a drug-usage evaluation of linezolid for January through April 2005. The drug-usage evaluation included prescriber information, duration, and indication for linezolid; patients with active linezolid orders were identified by concurrent computer printouts.

### Statistical Analyses

Statistical analyses were performed by using Epi Info 3.2.2 (CDC) and SAS v. 9.1 software (SAS Institute, Cary, NC, USA). Fisher exact test was used to compare categorical variables; the Kruskal-Wallis test was used to compare continuous variables. For univariate analysis, exact methods were used for 95% confidence intervals (CIs); for multivariate analysis, 95% Wald CIs were used. All p values were 2-sided.

## Results

**Figure 2:**
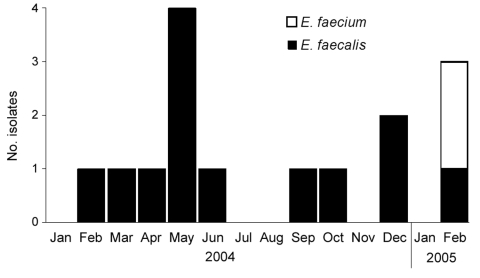
Occurrence of linezolid-resistant *Enterococcus faecalis* and *E. faecium* in hospital A, Tennessee, January 2004–February 2005 (N = 15).

For January 2004 through February 2005, a total of 15 LRE cases were identified (Figure 2): 2 (13%) were *E. faecium* and 13 were *E. faecalis* infections. Of the 15 case-patients, 12 (80%) were black and 8 (53%) were female; the median age was 54 years (range 38–74 years) (Appendix Table). Two of the case-patients had been admitted to the hospital from a nursing home. For 8 of the 15 patients, diabetes was a previous medical condition; 2 patients had previously required dialysis, and no patients had been transplant recipients. Eight patients had required care in a critical-care area before acquiring an LRE infection. Six (40%) case-patients died.

### Case-Control Study I

Sixty control patients with LSE clinical isolates were identified. Case-patients and LSE patients did not differ significantly by age, race, sex, immunocompromising conditions, or place of origin (e.g., nursing home) (Appendix Table). Of the enterococcal isolates from case-patients and controls, 13 (87%) and 53 (88%), respectively, were *E. faecalis*. LRE isolates were more likely to have been invasive than LSE isolates (8 [53%] vs. 12 [20%]; odds ratio [OR] 4.6; 95% CI 1.2–17.9).

Patients with LRE infection were more likely to die during their hospitalization than those with LSE infection (OR 9.3; 95% CI 1.8–51.2). Case-patients were hospitalized significantly longer than controls (median 35 days [range 1–127] for case-patients vs. 11 days [range 1–140] for controls; p<0.001). Case-patients were hospitalized for longer periods than control patients before the index culture (20 vs. 4 days; p<0.001) and after the index culture (19 vs. 9 days; p = 0.002).

During the preceding 12 months, LRE case-patients had received more cumulative days of antimicrobial drug treatment than LSE patients (median 58 vs. 18 days; p = 0.003). Compared with patients with LSE infection, patients with LRE infection had more frequently received linezolid therapy during the prior 14 days (27% vs. 5%; OR 9.7; 95% CI 1.3–76.8). This association remained valid for receipt of linezolid in the prior 2, 3, and 12 months. Patients with LRE infection, compared with patients with LSE infection, also had more cumulative days of linezolid therapy (median 15 vs. 0 days; p = 0.009); among those who had received linezolid, the median was 17.5 days versus 8 days (p = 0.06). Case-patients were less likely than control patients to have received vancomycin during the previous year. Apart from carbapenems, no other antimicrobial drugs were associated with LRE infection; carbapenems were usually prescribed together with linezolid.

Case-patients with LRE were also more likely than controls to have had a prior clinical isolation of MRSA (OR 13; 95% CI 3.0–60.4): 10 (67%) case-patients and 8 (13%) control patients had an MRSA infection during the 12 months before the index hospitalization. Of the 10 case-patients with a previous MRSA infection, 5 had received linezolid therapy (4 after isolation of MRSA) before their LRE infection.

Next, we stratified prior infection with MRSA by linezolid exposure. Among patients who had no linezolid exposure, prior MRSA infection remained strongly associated with subsequent LRE infection (OR 23.0; 95% CI 2.6–272.0). Conversely, when we stratified linezolid exposure by prior MRSA infection, the exposure was associated with LRE infection among those with no previous MRSA infection (OR 11.5; 95% CI 1.0–152.8). The association between prior MRSA infection and hospital stay before the index culture did not reach statistical significance (median 10 vs. 5 days; p = 0.2).

Case-patients with LRE infection were more likely than control patients to have spent time in 4 locations in hospital A before the index culture occurred. These locations were 3 specific critical care areas (a medical-surgical ICU, extended postoperative holding area, and ventilator rehabilitation unit; OR 11.0; 95% CI 2.1–61.4) and 1 orthopedic/neurosurgical ward (OR undefined). By multivariate analysis, using forward logistic regression, we identified the following as statistically significant predictors of LRE infection: prior isolation of MRSA (adjusted OR [AOR] 27; 95% CI 4.3–174), duration of hospitalization before index culture (AOR 1.1 per day; 95% CI 1.0–1.2), and duration of preceding linezolid therapy (AOR 1.1 per day; 95% CI 1.0–1.2).

### Case-Control Study II

To compare patients with LRVRE and LSVRE infections, we identified 7 case-patients and 28 controls. Case-patients and controls did not differ significantly by age, race, sex, immunocompromising conditions, instrumentation, or proportion of deaths. The length of hospitalization for case-patients compared with that for controls did not reach statistical significance (42 vs. 22 days; p = 0.15). During the previous 12 months, the cumulative days of total antimicrobial drug therapy, and of linezolid therapy specifically, did not differ significantly between case-patients with LRVRE and controls with LSVRE (median 19 vs. 6 days; p = 0.17).

### Prevalence Study

Nasal swab samples were obtained from 393 (93%) of the 424 hospitalized patients, and perirectal swab samples were obtained from 388 (92%). MRSA was isolated from 25 (6%) of the 393 nasal swab specimen cultures; linezolid-resistant *S. aureus* was not identified in the cultures. VRE was isolated from 51 (13%) of the 388 perirectal swab specimens, and LRE was isolated from 4 (1%); the 4 LRE isolates also were resistant to vancomycin. Of the 4 patients with LRE, 3 were located on 2 medical-surgical wards and 1 was located in a critical care area.

### Laboratory Studies

**Figure 3:**
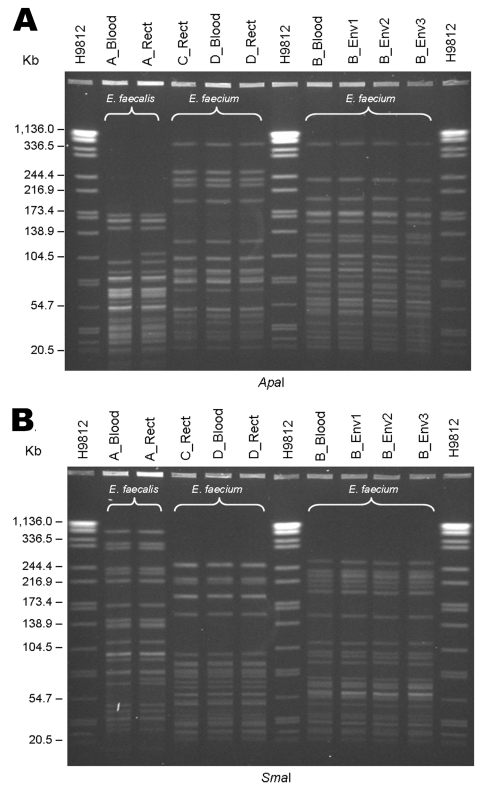
Pulsed-field gel electrophoresis of linezolid-resistant enterococci (LRE) isolates, hospital A, Tennessee. A) Digestion with *Apa*I. B) Digestion with *Sma*I. Isolates labeled A, B, C, and D refer to patients mentioned in the text. Blood, isolate from blood specimen culture; Rect, isolate from perirectal/rectal swab specimen culture; Env, environmental isolate; H9812, *S*. Braenderup H9812 strain (ATCC BAA-664) (*27*) used as size marker.

LRE isolates from 4 case-patients (patients A–D) and 3 environmental isolates from patient B’s room (sleep chair, blood-pressure cuff, and hospital bed on/off button) after routine cleaning were available for PFGE (Figure 3). The isolates from cultures of blood and rectal swab specimens from patient A (*E. faecalis*) were related but distinguishable (1 and 2 bands different for *Apa*I and *Sma*I, respectively). The environmental isolates (*E. faecium*) were indistinguishable from patient B’s blood culture isolate. A third pattern was shared between patients C and D; the isolates of *E. faecium* from blood and rectal swab specimens from patient D were identical.

### Antimicrobial Drug Use

**Figure 4:**
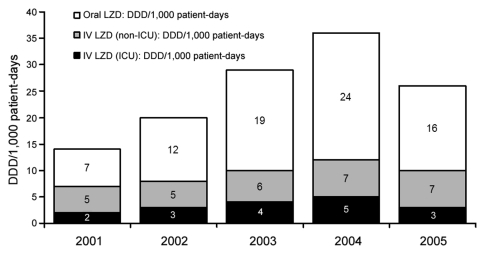
Linezolid (LZD) usage during 2001–2005 at hospital A, Tennessee. Use of oral and intravenous (IV) formulations is shown in defined daily doses (DDD)/1,000 patient days. Data for 2001 and 2005 do not include all 12 months (2001 includes data from October through December; 2005 includes data from January through February). ICU, intensive care unit.

Hospital A started using linezolid in April 2000; however, pharmacy purchase data were available only from October 2001 onward. DDD increased from 13/1,000 patient-days in 2001 to 35/1,000 patient-days in 2004 (Figure 4). Most linezolid doses were used outside critical care areas. The drug-usage evaluation identified 177 patients who received linezolid therapy (range of treatment duration 2–30 days). A total of 164 (93%) patients were prescribed linezolid by either infectious disease (118 [67% of 177 total patients receiving linezolid]) or critical care (46 [26% of 177 total patients receiving linezolid]) physicians. Four prescribers (3 infectious disease and 1 critical care) accounted for 144 (81%) patients; 1 prescriber accounted for 64 (36%) patients. Apart from restricting prescription of certain antimicrobial drugs to particular specialty groups, no antimicrobial drug stewardship program was in place.

### Infection-Control Policy Review

Patients with known VRE infection were placed on contact precautions. Patients with MRSA infection were placed on contact precautions while in critical care areas. Critical care–area staff performed surveillance cultures of urine and sputum on a weekly basis.

## Discussion

We describe risk factors and outcomes associated with a large hospital outbreak of LRE, an emerging pathogen. The licensing-to-resistance time interval for linezolid was brief. Linezolid use increased in response to increases in MRSA and VRE infection in this hospital community and probably because of the drug’s convenience compared with vancomycin (e.g., good bioavailability and no requirement for blood-level or renal-dose adjustment). Despite being restricted to use by infectious disease and critical care physicians, the linezolid DDD prescribed by hospital A increased nearly 3-fold in 3 years; most use was outside ICUs. In this investigation, we found that exposure to linezolid any time in the preceding 12 months and the increased cumulative days of linezolid use among case-patients suggest that antimicrobial drug pressure contributed to the emergence of multiple clones of LRE at hospital A. De novo resistance has been documented in several instances in which PFGE comparisons have been made between LSE and LRE isolates in the same patient after exposure to linezolid (*10*,*13*,*16*). Our controls consisted of patients with clinical LSE isolates; use of these controls can introduce selection bias, potentially overestimating the OR associated with prior exposure to linezolid (*29*,*30*). One could hypothesize that linezolid protects patients against having subsequent culture results positive for LSE and, thus prevents patients from becoming members of the antimicrobial drug (linezolid)–susceptible control group. The duration of any such potential protection is unknown. However, 4 (50%) LRE case-patients with linezolid exposure had a nonimmediate exposure (>30 days before index culture) to linezolid, compared with 2 (22%) LSE controls.

At hospital A, 8 (53%) of 15 patients with LRE infection had been exposed to linezolid; however, 7 (47%) had not been. This finding has been documented previously with nosocomial transmission of LRVRE in a transplantation unit among 7 patients, of whom 6 were linezolid-naive, and isolated from all had the same pattern on *Sma*I PFGE (*17*). In another study, 2 patients without linezolid exposure acquired LRVRE with identical PFGE patterns (*31*). A clonal outbreak was described by Dobbs et al. (*21*); only 6 (15%) of the patients had received linezolid before contracting LRVRE, and 17 (42.5%) were in a particular ICU before acquiring LRVRE. In our investigation, patient C was linezolid-naive and located in the same critical care unit as patient D (linezolid-exposed); their isolates were identical on PFGE. Isolates recovered from patient B (linezolid-exposed) and from multiple environmental samples from patient B's hospital room after routine cleaning were indistinguishable with 2 enzymes by PFGE, indicating possible patient-to-patient transmission through contaminated fomites or healthcare workers’ hands. This pattern was distinguishable from both the patient A pattern and patient C/D pattern. Patient-to-patient transmission is further supported by the clustering of exposures in time (January–February 2005) and space (3 specific critical care units and an orthopedic/neurosurgical ward).

A strong risk factor for LRE was prior MRSA infection. In 4 (27%) case-patients, linezolid was used to treat prior MRSA infection. However, the relationship between prior MRSA and LRE goes beyond this expected association of prior MRSA and linezolid exposure. Even among those with no linezolid exposure, MRSA was strongly associated with linezolid resistance (OR 23; 95% CI 2.6–272.0). This was confirmed on multivariate analysis (AOR 27). Prior MRSA infection therefore might also be a surrogate marker for patient-to-patient transmission of LRE or increased susceptibility to nosocomial infection.

In this outbreak, patients with LRE infection experienced more illness, were hospitalized longer, and were more likely to die than patients with LSE infection. We cannot determine whether the higher number of deaths were attributable to LRE; additional studies are required, including matching of controls to case-patients on severity-of-illness measures. In addition, our sample size and statistical power were limited by the number of cases of LRE and, in particular, LRVRE.

Infection-control efforts should focus on preventing infections and interrupting patient-to-patient transmission of multidrug-resistant organisms (MDROs) (*32*,*33*). Widespread rise in MDROs (e.g., MRSA) likely contributes to increases in linezolid prescription and LRE. Tracking or restricting linezolid use (e.g., for treatment of invasive VRE or MRSA) might reduce antimicrobial drug pressure and slow down emergence of LRE, which is critical because only a limited number of antimicrobial drugs are available to treat resistant gram-positive infections. Recently published guidelines (*34*) recommend use of additional interventions, such as active surveillance cultures and contact precautions, if either of the following 2 conditions is met: “1) the incidence or prevalence of MDROs are not decreasing despite the use of routine control measures; or 2) the first case or outbreak of an epidemiologically important MDRO (e.g., VRE, MRSA) is identified within a healthcare facility or unit. Facilities should continue to monitor the incidence of target MDRO infection and colonization; if rates do not decrease, implement additional interventions as needed to reduce MDRO transmission.” However, some hospitals might not implement active surveillance cultures because of concerns about potential delays in discharging colonized patients to nursing homes. Other obstacles are logistic (e.g., ensuring compliance rates >90% for surveillance cultures) and financial (e.g., patients cannot be charged for surveillance cultures, or insufficient infection-control resources might exist). These concerns and obstacles should be addressed; otherwise, the response to 1 emerging resistant infection will breed another emerging infection.

## Supplementary Material

Appendix Table Demographics, laboratory data, risk factors, and outcomes for case-patients with linezolid-resistant enterococci (LRE) and for control-patients with linezolid-sensitive enterococci (LSE) infection*
