# Supplementary material for: Response to Emerging Infection Leading to Outbreak of Linezolid-Resistant Enterococci
Source: Emerg Infect Dis. 2007 Jul;13(7):1024–30. doi: 10.3201/eid1307.070019 (PMC2878236; doi:10.3201/eid1307.070019)
Supplement: Appendix Table — Demographics, laboratory data, risk factors, and outcomes for case-patients with linezolid-resistant enterococci (LRE) and for control-patients with linezolid-sensitive enterococci (LSE) infection* [file 07-0019_appT-s1.pdf]

Appendix Table. Demographics, laboratory data, risk factors, and outcomes for case-patients with linezolid-resistant enterococci (LRE) and for control-patients with linezolid-sensitive enterococci (LSE) infection*

Variable	Case-patients with LRE† (N = 15)‡	Control patients with LSE† (N = 60)	Odds ratio (95% confidence interval)
Demographics			
Sex			
Male	7 (47)	19 (32)	
Female	8 (53)	41 (68)	0.5 (0.2–1.9)
Race			
White	2 (13)	16 (27)	
Black	12 (80)	41 (68)	1.9 (0.4–9.4)§
Other	0	1(2)	
Unknown	1 (7)	2 (3)	
Age, median years (range)	54 (38–74)	65 (20–93)	p = 0.09
Laboratory data			
Species information			
Enterococcus faecalis	13 (87)	53 (88)	0.9 (0.1–6.8)
E. faecium	2 (13)	7 (12)	
Source of index isolate			
Invasive site	8 (53)	12 (20)	4.6 (1.2–17.9)
Blood	7 (47)	12 (20)	
Peritoneal fluid	1(7)	0	
Urine	4 (27)	36 (60)	
Wound	2 (13)	6 (10)	
Catheter tip	1(7)	2 (3)	
Other	0	4 (7)	
Susceptibility, vancomycin resistant	7 (47)	11 (18)	3.9 (1.0–15.2)
Risk factors			
Antimicrobial drug treatment			
Mean no. agents used in previous 12 mo	13	6	p = 0.004
Median no. days used in previous 12 mo	58	18	p = 0.003
Linezolid treatment			
Use in prior 12 mo	8 (53)	9 (15)	6.5 (1.6–26.5)
Time interval (d) between any use and index culture			
14	4 (27)	3 (5)	9.7 (1.3–76.8)
30	4 (27)	7 (12)	4.2 (0.7–21.7)
60	7 (47)	7 (12)	7.3 (1.6–32.7)
90	7 (47)	8 (13)	6.4 (1.4–27.6)
365	8 (53)	9 (15)	6.5 (1.6–26.5)
No linezolid exposure	7 (47)	51 (85)	Referent

Length of treatment, determined using all patients, median no. d (range)	15 (0–32)	0 (0–25)	p = 0.009
Length of treatment, determined using only patients prescribed linezolid), median no. d (range)	17.5 (6–32)	8.0 (4–25)	p = 0.06
MRSA clinical isolate prior to index culture	10 (40)	8 (3)	13 (3.0–60.4)
Stratification of linezolid exposure by MRSA			
Prior MRSA (n = 10)			
Linezolid exposure	5 (50)	3 (38)	1.7 (0.2–16.8)
No linezolid exposure	5 (50)	5 (63)	
No prior MRSA (n = 5)			
Linezolid exposure	3 (60)	6 (12)	11.5 (1.0–152.8), FE = 0.02
No linezolid exposure	2 (40)	46 (88)	
Stratification of prior MRSA by linezolid exposure			
Linezolid exposure (n = 8)			
Prior MRSA	5 (63)	3 (33)	3.3 (0.3–37.6)
No prior MRSA	3 (38)	6 (67)	
No linezolid exposure (n = 7)			
Prior MRSA	5 (71)	5 (10)	23.0 (2.6–272.0)
No prior MRSA	2 (29)	46 (90)	
Hospitalization			
Median no. total days (range) hospitalized	35 (1–127)	11 (1–140)	p<0.001
Median no. days (range) hospitalized before index culture	20 (1–94)	4 (0–44)	p<0.001
Median no. days (range) hospitalized after index culture	19 (1–50)	9†(1–121)	p = 0.002
Location in hospital A during 90 d before index culture			
Any critical care area	8 (53)	13 (22)	4.1 (1.1–16.0)
In 1 of 3 specific critical care areas	6 (40)	4 (7)	11.0 (2.1–61.4)
Medical-surgical ICU	3 (20)	2 (3)	7.3 (0.7–92.0)
Extended postoperative holding area	3 (20)	1 (2)	14.8 (1.0–786.8)
Ventilator rehabilitation unit	4 (27)	1 (2)	21.5 (1.8–1073.4)
Orthopedic/neurosurgical ward	2 (13)	0	Undefined, Wald estimate = 95
Inpatient in hospital A during September 2004	2 (13)	0	Undefined, Wald estimate = 95
Inpatient in hospital A during January–February 2005	4 (27)	0	Undefined, Wald estimate = 222
Located in medical-surgical ICU (January)	2 (13)	0	Undefined, Wald estimate = 95
Located in ventilator rehabilitation unit (February)	2 (13)	0	Undefined, Wald estimate = 95
Instrumentation (receipt of the following)			
Ventilator	8 (53)	10 (17)	5.7 (1.5–23.3)
Foley catheter	11 (73)	27 (45)	3.4 (0.9–14.3)

Peripheral inserted central catheter	10 (67)	29 (48)	2.1 (0.6–8.3)
Central venous catheter	4 (27)	14 (23)	1.2 (0.2–4.9)
Chest tube	3 (20)	0	Undefined, FE = 0.007
Permanent central venous catheter	2 (13)	8 (13)	1.0 (0.1–5.9)
Arterial line	0	1 (2)	0 (0–156)
Dialysis catheter	1 (7)	5 (8)	0.8 (0–7.9)
Balloon pump	0	0	–
Pulmonary artery catheter	0	0	–
Intraabdominal surgery	1 (7)	3 (5)	1.4 (0–18.4)
Other surgery	3 (20)	10 (17)	1.3 (0.2–5.9)
Outcome			
Death	6 (40)	4 (7)	9.3 (1.7–52.4)
Median no. total days (range) of hospitalization	35 (1–127)	11 (1–140)	p<0.001
Median no. days (range) hospitalized after index culture	19 (1-50)	9 (1–121)	p = 0.002

*MRSA, methicillin-resistant *Staphylococcus aureus*; ICU, intensive care unit; FE, Fisher exact test (as opposed to p value).

†Data are no. (%) unless otherwise noted in first column.

‡N = 15, unless otherwise indicated in first column.

§Black compared with all other races.

¶Invasive compared with noninvasive isolates.
